# Implementing a home-based personalised cognitive rehabilitation intervention for people with mild-to-moderate dementia: GREAT into Practice

**DOI:** 10.1186/s12877-022-03705-0

**Published:** 2023-02-13

**Authors:** Linda Clare, Aleksandra Kudlicka, Rachel Collins, Suzannah Evans, Jackie Pool, Catherine Henderson, Martin Knapp, Rachael Litherland, Jan Oyebode, Robert Woods

**Affiliations:** 1 University of Exeter Medical School, Exeter, EX1 2LU UK; 2 NIHR Applied Research Collaboration South-West Peninsula, Exeter, UK; 3 Betsi Cadwaladr University Health Board, Bangor, UK; 4 Glyndwr University, Wrexham, UK; 5 QCS Quality Compliance Systems, London, UK; 6 Care Policy and Evaluation Centre, London School of Economics and Political Science, London, UK; 7 Innovations in Dementia CIC, Exeter, UK; 8 Centre for Applied Dementia Studies, Bradford University, Bradford, UK; 9 Dementia Services Development Centre, Bangor University, Bangor, UK

**Keywords:** Alzheimer’s disease, Vascular dementia, Parkinson’s disease, Dementia with Lewy bodies, Activities of daily living, Functional ability, Disability, Reablement, Carers, Occupational therapy

## Abstract

**Background:**

Evidence-based rehabilitative interventions, if widely implemented, could equip people with dementia and their families to manage life with the condition and reduce the need for health and care services. The aim of this translational study, building on evidence from the GREAT randomised controlled trial, was to develop a foundation for implementing the GREAT Cognitive Rehabilitation intervention in community-based services for people with mild-to-moderate dementia.

**Methods:**

Key elements of the implementation strategy were identifying and supporting managerial and clinical leadership, conducting collaborative planning and target-setting, training and supporting practitioners, and providing external facilitation. We developed implementation plans with, and trained staff in, 14 organisations. We subsequently worked closely with 11 of these, 10 National Health Service organisations and one private home care provider, to support practitioners to deliver GREAT Cognitive Rehabilitation over a 12-month period. Outcome evaluation examined the perspectives of local steering group members, practitioners and service users, and the reach, effectiveness and cost of the intervention.

**Results:**

Implementation was disrupted by the COVID-19 pandemic, but six organisations completed at least six months of intervention delivery. Forty-one practitioners, mainly occupational therapists, provided the intervention, and 54 people with dementia completed a course of GREAT Cognitive Rehabilitation. Goal attainment by people with dementia exceeded levels of improvement seen in the original trial. People with dementia, carers, practitioners and steering group members all evaluated the intervention positively, and economic analysis indicated that the intervention could be provided at modest cost. However, we identified a range of mainly organisational barriers that impeded implementation and limited the potential for sustainability.

**Conclusions:**

GREAT Cognitive Rehabilitation benefits people with dementia, can be delivered effectively at modest cost in routine services, and is viewed positively by people with dementia, family carers and practitioners. To fully realise these benefits and achieve widespread and sustainable implementation, however, requires sufficient resources and a reorientation of service priorities towards preventive and rehabilitative approaches.

**Trial Registration:**

National Institute for Health Research (NIHR) Central Portfolio Management System, registration number 38994.

**Supplementary Information:**

The online version contains supplementary material available at 10.1186/s12877-022-03705-0.

## Introduction

Dementia is a progressive syndrome characterised by gradual decline in cognitive and functional ability and capacity for independent living. There are currently no disease-modifying treatments. Globally there are over 55 million people living with dementia. The number is forecast to rise to over 150 million by 2050 [1, 2]. This will lead to an increase in need for and utilisation of health and social care services as well as support from families and friends. Most people with mild-to-moderate dementia live in the community, supported by unpaid family carers. In this stage of dementia, progressive impairment in cognition affects the ability to carry out complex activities, leading to an increased need for support in order to maintain participation in everyday life [3]. Evidence-based interventions designed to equip people with mild-to-moderate dementia and their carers with the ability to manage life with the condition could play an important role in addressing some of the need for health and social care support [2, 4–6]. Even in high-income countries such as the UK, however, provision of post-diagnostic support for people in the mild-to-moderate stages of dementia and their families remains limited in scope and quality [7]. There is little opportunity to access evidence-based psychosocial or rehabilitative interventions [8].

One such intervention that could be impactful if widely implemented is cognitive rehabilitation (CR). CR is a goal-oriented individualised behavioural therapy addressing the impact of cognitive impairment on everyday functioning that has been adapted for people with mild-to-moderate dementia [9]. CR is designed to support people with dementia in managing their everyday activities by facilitating behaviour change that builds on their strengths and enables them to function at their optimal level and sustain an appropriate level of independence; it is conducted in the home setting, with carers fully involved and supported where possible [10].

The main research evidence for the benefits of this approach for people with Alzheimer’s, vascular, mixed and Parkinsonian dementias comes from single-site and multi-centre trials conducted in the UK and France. These trials have demonstrated improvements in functional ability in relation to goals targeted in the intervention [11–14], and delayed institutionalisation [15]. Based on this evidence, in the UK the National Institute for Health and Care Excellence (NICE) recommends that services consider offering CR to support functional ability for people with mild-to-moderate dementia [16], and the Memory Services National Accreditation Programme (MSNAP) lists providing CR following a diagnosis of dementia as one of the goals that memory services should aspire to meet [17]. However, the approach is not offered routinely, suggesting that focussed efforts might be required to support its integration into practice. The GREAT into Practice (GREAT-iP) project described here built on the UK GREAT trial [11], and was funded by Alzheimer’s Society in the UK as a translational study aimed at developing the foundation for widespread implementation of the evidence-based GREAT CR intervention into service provision for people living with mild-to-moderate dementia.

In the final stages of the UK GREAT trial, pilot work in the form of a small-scale feasibility study focused on how to implement GREAT CR in routine service provision at three National Health Service trial sites [12]. This informed development of the GREAT-iP implementation strategy, which was guided by a comprehensive theoretical framework. The Knowledge-into-Action Process Framework [18] provided the domains to be covered: evidence, context, methods, adoption and outcome. To operationalise these, as no single implementation science model covered all five domains, we drew on several models: the Ottawa Model of Research Use [19], the Promoting Action on Research Implementation in Health Services Framework [20], the Stetler Model of Research Utilization [21], and the taxonomy of implementation outcomes [22]. The expected mechanism for achieving adoption and effective implementation involved three core components: internal and external facilitation, collaborative planning and flexible tailoring, and identifying and managing barriers. Underpinning elements were effective communication of the evidence and rationale for the intervention, and a sound understanding of the service contexts and practitioners involved.

We applied this strategy to conduct a translational implementation study addressing the following questions:

- Implementation objectives
  How readily can community-based services engage in providing GREAT CR?
  What barriers and facilitators affect the implementation of GREAT CR?
  What is the potential for scaling up the implementation?
- Intervention objectives
  Is GREAT CR effective when delivered as part of routine services?
  What are the views of people with dementia, carers, and practitioners about GREAT CR?
  What is the per-person cost to services of delivering GREAT CR?

## Method

### Design

The project focused on implementation of GREAT CR in routine services and included a research component to evaluate the effectiveness of the implementation strategy. The evaluation was approved by Wales Research Ethics Committee 5, reference 18/WA/0217. Informed consent was obtained from all study participants: representatives of partner organisations, practitioners working within those organisations, and people with dementia and carers receiving the intervention as service users. The study was registered on the National Institute for Health Research (NIHR) Central Portfolio Management System, registration number 38994. The project started on 1^st^ January 2018 and was intended to run for 36 months, until 31^st^ December 2020; the end date was subsequently extended to 31^st^ July 2021. The work was conducted with involvement of people living with dementia and carers; two individuals living with dementia were part of the study leadership team, and three Alzheimer’s Society research volunteers with caregiving experience acted as ‘critical friends’, monitoring progress and offering advice and feedback.

### Context

The implementation, conducted in England and Wales, targeted three types of community-based service provider: state-funded National Health Service (NHS) organisations each providing specified health services free of charge at the point of delivery to the population of a defined geographical area; social care departments of local authorities (municipalities) whose services incur a cost for people with assets above a modest level; and non-state-funded home care businesses offering defined in-home support for paying customers on either a for-profit or non-profit basis.

### Characteristics of implementation sites

We aimed to work with 15 organisations; this number reflected what could feasibly be achieved with available funding. Organisations were eligible if they had at least one staff team providing home-based services to people living with dementia. Potential partner organisations were identified through direct contacts, expressions of interest following dissemination of GREAT trial findings, and the NIHR register. We proposed to train up to 12 staff members in each organisation; as resources allowed for one initial training course per organisation, this number reflected the need to provide an interactive group training experience. Staff were eligible to participate if they worked directly with people with dementia. To compensate for time required for the evaluation, each organisation was offered funds equivalent to one half-day per week of staff time at NHS Band 6 payment rates for six months; implementation costs were absorbed by the organisations.

### Characteristics of service users receiving the intervention

The study team recommended that, when identifying people with dementia to receive CR, practitioners should include people with a diagnosis of Alzheimer’s, vascular or mixed dementia or one of the Parkinsonian dementias, and in the mild-to-moderate stage as indicated by a Mini-Mental State Examination [23] score of 18 and above or equivalent. This was based on evidence from the GREAT [11] and CORD-PD [14] trials. Within these parameters, guidance was offered regarding the characteristics of people with dementia who responded particularly well in the GREAT trial [11]: relatively recently diagnosed, able to engage in at least some daily activities and hold a conversation about these with the practitioner, keen to reduce the impact of cognitive impairment on daily life, reasonably active, no complex physical or psychological co-morbidities, and supported by a carer who could encourage between-session practice.

### Implementation strategy

The implementation strategy covered the five domains of the Knowledge-into-Action Process Framework [18]: evidence, context, methods, adoption and outcome. The main mechanisms by which the strategy was intended to work were identifying and supporting managerial and clinical leadership within organisations, conducting collaborative planning and target-setting, and training and supporting practitioners. The project team provided external facilitation with the expectation that this would be gradually faded out towards the end of the 12-month implementation period as organisations became equipped to sustain the approach. We planned the implementation in two waves so that learning from the first wave could be incorporated in the second wave. See Additional Text 1 and Additional Table 1 for further details. To operationalise the strategy, we used a structured implementation planning tool to collaboratively develop and agree a tailored implementation plan for each organisation.

The implementation phase for each partner organisation began with a two-day foundation-level training course to prepare participating staff to deliver the intervention. Following this, staff were to begin offering the intervention, supported by monthly group supervision sessions with the external facilitator. Intervention delivery was to continue for 12 months as part of the project. Advanced-level training was to be made available for practitioners who gained sufficient experience at foundation level. The intention was that advanced-level training would equip at least some staff in each organisation to support others in delivering the intervention, allowing for a gradual withdrawal of the external facilitator and promoting sustainability. A train-the-trainer course was also envisaged. The external facilitator, together with another member of the project team, conducted a formal mid-way meeting with steering group members to review progress, problem-solve regarding any barriers encountered, and explore and plan for future sustainability. We aimed to examine sustainability by following the first wave organisations for a further 12 months, and the second wave organisations for a further six months, after the end of the implementation period.

### Description of the intervention

GREAT CR involves a course of individual sessions conducted in the home setting. Realistic and personally-meaningful goals are agreed in collaboration with the CR practitioner. The practitioner, person with dementia and carer then collaboratively develop and implement a plan to support behaviour change leading to goal attainment, addressing the identified needs using evidence-based rehabilitative strategies. These include both enhanced learning techniques and compensatory approaches, such as environmental modification and introduction of memory aids. The personalised focus allows for considerable flexibility, ensuring that individual needs are directly addressed, and plans can be adapted as necessary to support goal attainment. At a behavioural level, rehabilitation strategies establish new behavioural routines and provide an experience of success in tackling challenges encountered in everyday situations; this in turn encourages development of a solution-focused approach that can be applied in other situations, and helps to build or rebuild confidence, which can lead to wider benefits. Neuroimaging findings suggest changes are underpinned by increased activation in relevant compromised brain areas [24].

In the GREAT trial [11, 12], CR was delivered by a single professionally-qualified practitioner at each site according to a pre-defined protocol that specified the number of sessions (up to 10, plus four maintenance sessions), suggested a broad structure for practitioners to follow, and incorporated additional elements such as anxiety management. In routine practice, CR could potentially be applied more flexibly as regards number of sessions and practitioner skill-mix. Pilot work conducted at the end of the GREAT trial demonstrated that gains could be achieved with six sessions, and occupational therapy (OT) assistants could deliver the intervention effectively under supervision by a qualified professional, thus enhancing the potential for affordable implementation. Therefore, we worked with stakeholders to consider what adaptations should be made to intervention delivery. The following parameters were recommended:

- Offering an average of six one-hour sessions with flexibility to tailor the number to individual needs.
- Working on one goal, or possibly two if time allowed, and focusing on the types of rehabilitation strategy that were directly relevant to the chosen goals.
- Augmenting the goal-oriented work with additional components, such as anxiety management, only where specifically indicated, rather than including these components in all cases as was done in the GREAT trial.
- Allowing flexibility in delivery so that qualified practitioners could either deliver the intervention themselves or involve unqualified assistants or technicians in conducting planned sessions.

We prepared a range of resources to support implementation and intervention delivery. See Additional Text 2 for details.

The primary intervention outcome in GREAT CR is goal attainment, quantified using the Bangor Goal-Setting Interview (BGSI) [25]. This employs a simple Likert-style scale with a rating capturing the perceived level of functioning related to each specific goal or area of need. Ratings of functioning are made initially and at follow-up by the person with dementia, by the carer where available and the CR practitioner, with changes over time indicating the extent of goal attainment; the practitioner also makes a percentage rating of the degree of goal attainment. Additionally, the person with dementia makes an initial rating of readiness to change, which helps ensure that the goals selected are meaningful and important. For the purposes of this study, we developed a short, simplified version of the Bangor Goal-Setting Interview (BGSI-S) [26].

### Evaluation

We planned to collect process and outcome data at organisation and practitioner level, assess outcomes for service users, and examine per-person costs to the organisation.

#### Organisational level

Quantitative indicators of implementation success were derived from assessing outcomes against targets identified in the implementation plan. We planned to gather qualitative data about the implementation process, focusing in particular on leadership, organisational barriers and facilitators, and potential for sustainability, through interviews at the end of the 12-month implementation phase with a purposively-sampled set of up to 24 local steering group members representing organisations with good and poor implementation outcomes; for the topic guide, see Additional Text 3. We planned to re-interview up to eight Wave 1 interviewees after 12 months to explore the extent to which progress was sustained.

#### Practitioner level

Practitioners were invited to complete an online survey at the end of the 12-month implementation phase covering their perspectives on the training and support they received, the intervention, and any barriers and facilitators affecting implementation. To capture more in-depth process data, interviews were conducted with a sub-group who expressed willingness to be interviewed; see Additional Text 3 for the topic guide.

#### Service user level

We explored quantitative indicators of intervention outcomes and service user experience and satisfaction. To characterise the population receiving the intervention at initial assessment, practitioners recorded anonymised demographic information for the person with dementia and carer, and used the Global Deterioration Scale [3] to describe severity of impairment and an adapted version of the Functional Activities Questionnaire [27, 28] to describe the functional ability of the person with dementia. Goal-setting was conducted using the BGSI-S [26]. Ratings of current functioning in relation to each goal were made using a 10-point scale (1 – unable to do or not currently doing and 10 – able to do well with no difficulty) by the person with dementia, the practitioner and the carer where available. Outcomes of the intervention were assessed by comparing the BGSI-S initial and post-intervention ratings of functioning for the identified goals. Practitioners completed a record sheet providing brief details of the goals addressed and strategies used. The person with dementia and the carer each evaluated their experience of GREAT CR via a brief anonymous questionnaire provided by the practitioner in the final session, which they returned by post to the Project Manager in a pre-paid envelope.

#### Evaluation of costs

We aimed to estimate the per-person costs of GREAT CR for people with dementia based on parameters observed during the implementation. Where possible we sought information about practitioner qualifications and grade, about the number and duration of CR sessions they provided, and whether they involved an unqualified assistant in intervention delivery. Based on what we learned we devised a series of different skill-mix scenarios that were representative of the practices observed across sites and used appropriate sources to calculate unit costs.

### Data analysis

Quantitative indicators of implementation and intervention outcomes, and costs of providing the intervention, were reported descriptively. Responses to open-ended survey questions were categorised using content analysis. Framework analysis [29] was employed to analyse information from qualitative interviews. The Framework method is a form of thematic analysis, suitable for semi-structured interview data, in which data are systematically categorised, coded and organised using a matrix to summarise the content by case (in this instance, by participant) and by code. This facilitates a process of comparison across and within cases that leads to the identification and refinement of themes, whether deductive (pre-selected based on prior literature, as in this case) or inductive (generated from the data). We developed a thematic framework based on constructs outlined in the theoretically derived implementation model used in the project, which were also included in the Consolidated Framework for Implementation Research [30]. These constructs were reflected in the broad categories addressed in each of the interview topic guides (see Additional Text 3). An independent researcher not otherwise involved in the project developed code books from initial analyses which were reviewed with study team members and then applied to the remaining interviews. Codes, themes and findings were iteratively discussed with other study team members at each stage, and any differences of opinion regarding the coding process or interpretation of findings were resolved through discussion.

## Results

### Characteristics of implementation sites

**Fig. 1:**
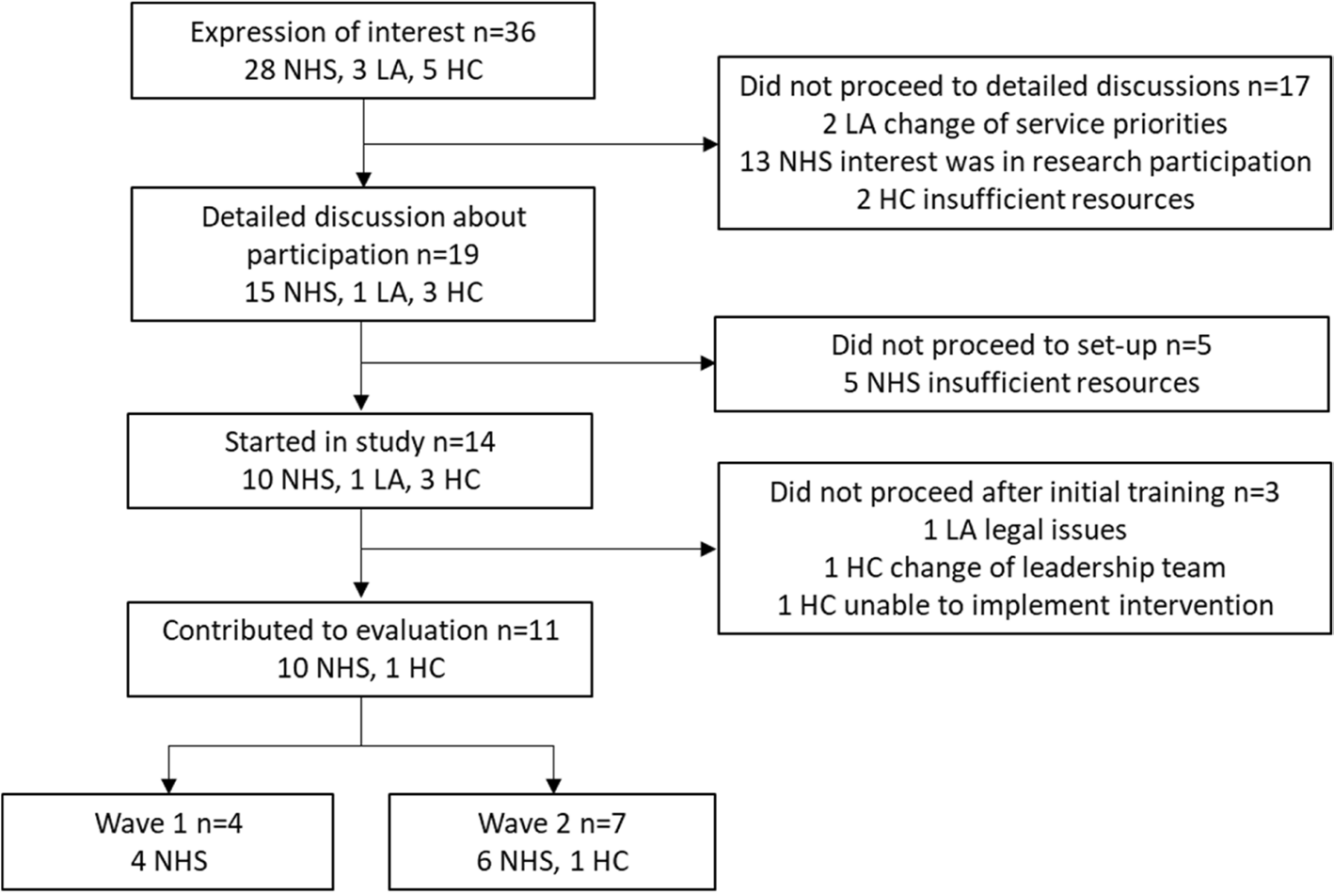
Flowchart summarising identification and engagement of partner organisations *National Health Service organisation, NHS; local authority, LA; home care provider, HC*

Of 36 organisations initially expressing interest, two local authorities subsequently reported changes in priorities, two home care providers were insufficiently resourced, and 13 NHS organisations were seeking funded involvement in research studies. Nineteen organisations engaged in detailed discussion. Five NHS organisations did not proceed due to perceived insufficiency of resources and staffing. Therefore, 14 organisations developed and agreed an implementation plan. These were 10 NHS organisations, three home care businesses and one local authority. Following staff training, one of the home care businesses failed to implement the intervention, and another withdrew due to an unexpected change in the entire senior management team. In addition, the legal department of the local authority objected to the wording of study documents required by the Health Research Authority (HRA), making it impossible to proceed; the HRA, through ethical review, protects and promotes the interests of people involved in health and social care research in England. This left 11 organisations, 10 NHS services and one home care business, that participated in the implementation and evaluation based on agreed implementation plans. Four of the organisations participated in Wave 1 and seven in Wave 2. During Wave 2, the implementation was disrupted by the COVID-19 pandemic. See Fig. 1 for a flowchart summarising identification and engagement of partner organisations.

Ninety-four staff in the 11 organisations received the two-day foundation-level training, and 88 provided immediate feedback, which was uniformly positive with some constructive suggestions for enhancement. The majority of trainees were occupational therapists (OTs; *n* = 54), OT assistants or OT technicians (*n* = 7). Other staff groups trained were clinical psychologists (*n* = 7), assistant psychologists (*n* = 3), nurses (*n* = 6) and support workers or healthcare assistants (*n* = 14); the professional background of three practitioners was not recorded. A small number of managers and supervisors, who were not expecting to deliver the intervention but wished to understand it in order to support their practitioners, additionally attended some training sessions. Forty-one practitioners went on to provide the intervention.

### Characteristics of service users receiving the intervention

**Fig. 2:**
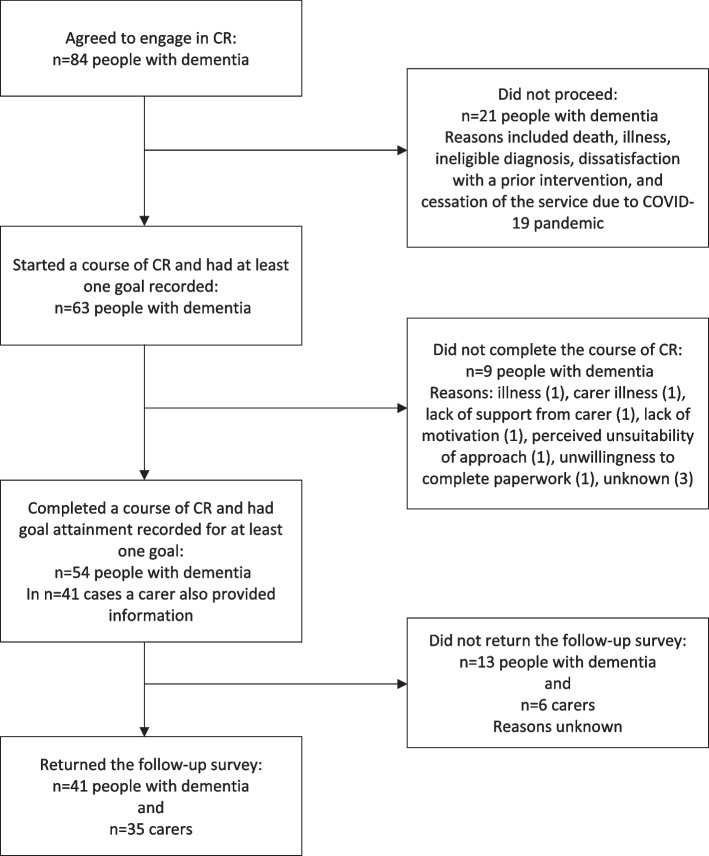
Flowchart summarising participant engagement

Eighty-four people with dementia agreed to receive the intervention. Twenty-one did not proceed; reasons were not available in all cases, but included death, illness, ineligible diagnosis, being unhappy about a previous intervention, and cessation of services due to the COVID-19 pandemic. Sixty-three people with dementia started a course of CR and had an initial rehabilitation goal recorded. Nine did not complete the course of CR; reasons, available for 6, were illness, carer illness, lack of support from carer, lack of motivation, perceived unsuitability, and too much paperwork. Fifty-four people (average age 76.02, 55% male) completed a course of CR, typically consisting of six hour-long sessions. See Fig. 2 for a flowchart summarising participant engagement.

Those who completed a course of CR were mostly white British, and nearly one-third had no formal educational qualifications. The most common diagnosis was Alzheimer’s disease. Information was available for 41 carers who contributed; they were predominantly white British, with an average age of 68 years and a broad range of educational backgrounds. They were mostly female, and either spouses (58.6%) or children (31.0%) of, and mostly (72%) co-resident with, the person with dementia. Characteristics of the people with dementia and carers are summarised in Additional Table 2.

### Implementation outcomes

Of the 11 organisations, nine set targets for the number of practitioners providing the intervention (71 in total, an average of seven per organisation, range 3–14) and 10 set targets for the number of people with dementia receiving the intervention (266 in total, an average of 26 per organisation, range 12–44). Other organisation-specific targets included increasing the focus on providing psychosocial interventions [16], or demonstrating the potential of such interventions to commissioners. Achievements in relation to targets are summarised in Table 1. The number of practitioners attending foundation-level training was 94, but only 41 subsequently provided CR. The number of people with dementia who started a course of CR (indexed by having a therapy goal recorded) was 63. The discrepancy between target and actual numbers was partly attributable to the impact of the COVID-19 pandemic. Courses of CR were reportedly provided outside the evaluation for some people with dementia unwilling to participate in the research element of the project, and some practitioners reported incorporating elements of CR into other work.

**Table 1:** Summary of implementation outcomes

ID	Wave	Type	Weeks of CR delivery (% of target)	Practitioners to be trained and deliver CR (target)	Practitioners trained	Practitioners providing ≥ 1 course of CR within the evaluation	Target achieved %*	People with dementia to receive CR (target)	People with dementia consented	People with dementia setting a goal	People with dementia completing a course of CR	Target achieved %^
1	1	NHS	58 (112%)	3	4	3	100%	12	12	10	8	83%
4	1	NHS	59 (113%)	14	13	10	71%	30	15	14	10	47%
8	1	NHS	57 (110%)	5	8	3	60%	16	12	11	11	69%
5	1	NHS	32 (62%)	7	6	4	57%	16	3	3	2	19%
9	2	NHS	28 (54%)	11	10	4	36%	44	12	12	12	27%
15	2	NHS	28 (54%)	5	11	2	40%	20	7	5	5	25%
14	2	NHS	18 (35%)	-	8	4	-	24	5	4	3	17%
10	2	NHS	12 (23%)	7	7	1	14%	28	1	1	1	4%
12	2	NHS	12 (23%)	11	13	2	18%	44	8	2	1	5%
11	2	NHS	12 (23%)	8	9	0	0%	32	8	0	0	0%
13	2	HC	6 (12%)	-	5	1	-	-	1	1	1	-

^*^target vs actual number of practitioners providing CR; ^target vs actual number of people with dementia receiving CR (indexed by setting a goal). Cognitive rehabilitation, CR; National Health Service organisation, NHS; home care provider, HC

For Wave 1 organisations, training took place between July and September 2018. CR delivery started following training and continued for up to 59 weeks, completing between August and December 2019. In one organisation (#5) there was a six-month delay between training and the start of CR delivery due to major staffing changes, and adaptations were made by the study team to accommodate this. The Wave 2 organisations received training between September and November 2019 and were expected to continue providing CR for up to 52 weeks, but due to the COVID-19 pandemic, all had to cease intervention delivery in early March 2020. Two (#9 and #15) were able to resume provision in November 2020 and continue until March 2021. For the three Wave 1 organisations that had the full 12-month delivery period, achievement of targets ranged from 60–100% for practitioners providing, and from 47–83% for people receiving, the intervention. For the three organisations that had approximately half of the expected period, achievement ranged from 36%-57% for number of practitioners providing, and 19–27% for number of people receiving, the intervention. The five organisations whose intervention delivery period was 18 weeks or less had limited opportunity to demonstrate outcomes in relation to targets; however, three each managed to deliver one course of CR.

Due to the COVID-19 pandemic, we were unable to conduct mid-way reviews with Wave 2 organisations or a follow-up exploration of sustainability. However, nine practitioners completed all elements of the foundation-level training and attended advanced-level training. Four subsequently completed all the advanced-level requirements.

### Implementation processes

Information about implementation processes and about barriers and facilitators to successful implementation was gathered from senior staff who served as steering committee members and practitioners through survey responses, interviews, and mid-way review meetings.

#### The senior staff perspective

The senior staff perspective on the implementation was explored in mid-way review meetings and through telephone interviews with six steering group members from five organisations conducted after the end of the intervention delivery period; five interviewees were senior clinicians in leadership roles, and one was a research manager. One of the clinicians also contributed as a CR practitioner. They represented organisations with varying degrees of success in achieving implementation targets (#4, #8, #9, #11 and #14; see Table 1). A summary of findings from the senior staff interviews, with example quotes, is provided in Additional Table 3 Themes covered in the senior staff interviews were:

- Outcomes of introducing CR – the perceived benefits for people with dementia, carers and staff teams.
- Implementation processes – the organisational challenges that made it hard to implement the intervention effectively despite high levels of enthusiasm and good will.
- Prospects for sustainability – uncertainty over whether it would be possible to continue delivering the intervention in the future.

All the senior staff interviewed described positive impacts for their organisations. CR was thought to encourage a more person-centred and creative approach in planning and delivering interventions, leading to better outcomes for people with dementia and carers, and improved morale and confidence among staff. Positive features of the intervention included the flexibility to accommodate individual needs and preferences, and the legitimacy resulting from the perceived strength and quality of the evidence-base.

Despite these positive views, all senior staff interviewed acknowledged that the implementation outcomes could have been better. Most of the barriers identified and discussed in the senior staff interviews were organisational. The primary external influence was local health service commissioning, which did not always support provision of psychosocial interventions. Within organisations, contextual factors related to culture, structure, leadership, organisational change and resources. In organisations where nihilistic views about dementia were prevalent or there was strong adherence to a medical model, it was difficult to secure resources and referrals for psychosocial interventions. Across all organisations, dementia care focused primarily on complex needs and responding to crises, and preventive or rehabilitative work was not part of practitioner remits. Senior managers often did not provide sufficient leadership to support a change in approach. In those cases where motivated managers and committed staff champions ensured that CR was kept on the agenda in their organisations and informed managers and colleagues about it, for example by giving presentations at meetings and using case examples, this helped to gain support and stimulate referrals. Across all services, there were high levels of staff absence and job vacancies, and instances of organisational change disrupting referral pathways. This meant that, contrary to the agreed implementation plans, practitioners were required to deliver CR on top of their usual caseload, and hence had limited opportunity to develop confidence in providing the intervention.

All six senior staff were keen for their organisation to continue providing CR and were trying to make plans for taking the work forward. However, there was uncertainty about support from senior managers and concern that service priorities specified by commissioners might preclude the potential to offer CR in the future.

### The practitioner perspective

Understanding of the practitioner perspective was derived from survey responses and interview data. Twenty-four practitioners completed an online survey. Their characteristics are summarised in Additional Table 4; they were mostly white females with a background in occupational therapy. Responses to the closed questions are shown in Additional Fig. 1, and the analysis of open-ended responses, with example quotes, is summarised in Additional Table 5. We intended to conduct follow-up interviews with practitioners in partner organisations with a range of implementation success, but in practice we interviewed practitioners from all four Wave 1 organisations, seven in total. We were not able to interview practitioners in Wave 2 organisations due to COVID-related disruption. A summary of the findings from practitioner interviews, with example quotes, is provided in Additional Table 7. Themes covered in the practitioner interviews were:

- Application of CR – the benefits for people with dementia, carers and practitioners themselves
- Targeting of CR – understanding what characteristics indicated that someone was likely to benefit from the approach
- Factors affecting delivery of CR – the importance of gaining confidence, identifying people likely to benefit, and having enough time and resources to provide a truly personalised intervention
- Sustainability of CR – whether and how delivery of CR would continue in the future.

Practitioner perceptions of the intervention were positive. Most practitioners responding to the survey (22, 92%) thought the intervention was beneficial for people with dementia and would readily recommend it. Most (20, 83%) felt the training prepared them well, felt well-supported in providing the intervention (17, 71%), and found the skills they gained useful (22, 92%). In the interviews, practitioners were consistently positive about the benefits of the intervention while acknowledging that for some people complex needs and circumstances, especially physical ill-health, could impact on effectiveness. CR both achieved specific goals and promoted wider benefits such as restoring confidence, facilitating independence, instilling hope, and reducing carer burden. Practitioners attributed benefits to the person-centred and individualised nature of the intervention, the effectiveness of the techniques, the development of self-management capability, and positive engagement of carers.

Lack of time was the greatest barrier for practitioners, making it difficult to get to know the person with dementia sufficiently to deliver a personalised intervention, and in some cases leading to low attendance at supervision sessions provided by the external facilitator. The other main barrier for practitioners related to self-efficacy. Practitioners needed to develop confidence through experience of delivering CR and participating in supervision, and then access further training to enhance their skills and capacity to support others. Practitioners found it helpful to gain peer support by teaming up with other practitioners and to involve assistants or technicians in providing some of the sessions. Getting started with delivering CR could be particularly challenging, especially if there was a gap between attending training and first use of the approach. This concern was addressed in Wave 2 by asking practitioners to identify potential recruits prior to training; this proved effective in enabling CR delivery to start immediately. Considering these challenges and the general organisational context, fewer than half of the practitioners surveyed (10, 42%) believed their organisation could sustain delivery.

### Intervention outcomes

All participants with dementia for whom information was available worked on one goal, and 15 (28%) worked on a second. The average increase in ratings of goal attainment on the BGSI-S 0 -10 scale was 4.20 points for people with dementia, 4.37 for carers and 4.80 for CR practitioners. Almost all participants made some progress toward achieving their first goal, and more than half fully achieved it. Goals mainly related to managing everyday activities, tasks and situations or to using appliances, devices and the internet. Intervention outcomes and details of the targeted goals are summarised in Table 2. No harms or unintended effects were identified. Fidelity to delivering the core components of the intervention was examined through review of identified goals and rehabilitative strategies applied; the goals addressed were consistent with trial data and study guidance, and therapists employed the expected range of rehabilitative strategies. Case reports submitted for completion of foundation-level and advanced training requirements demonstrated good fidelity.

Survey responses (see Table 2) were received from 41 of the people with dementia who received a course of CR, and 35 carers; 93% of people with dementia and 100% of carers found CR very or somewhat useful, and almost all said they would recommend it to others. Responses to open-ended questions are summarised in Additional Table 6, with example quotes. People with dementia indicated that CR supported their everyday functioning and increased confidence and independence. Carers appreciated the opportunity to learn strategies for supporting their relative and saw how achieving a specific goal could lead to wider benefits.

**Table 2:** Summary of intervention outcomes

Rating	Rated by	Goal 1			Goal 2		
		N	Mean (SD)	Range	N	Mean (SD)	Range
(a) Intervention outcomes as rated by people with dementia, carers and CR practitioners							
BGSI-S Initial Readiness to change	Person with dementia	54	7.89 (2.10)	2–10	14	8.61 (1.94)	4–10
BGSI-S Initial Attainment rating	Person with dementia	53	3.84 (2.46)	1–10	14	4.82 (2.37)	1–9
BGSI-S Initial Attainment rating	Carer	44	3.20 (2.39)	1–10	12	4.25 (2.14)	1–9
BGSI-S Initial Attainment rating	CR practitioner	53	3.36 (2.30)	1–10	13	4.23 (1.88)	1–8
BGSI-S Post-intervention Attainment	Person with dementia	50	7.97 (2.14)	0–10	12	7.04 (2.60)	2–10
BGSI-S Post-intervention Attainment	Carer	41	7.54 (2.49)	0–10	10	7.30 (2.16)	3–10
BGSI-S Post-intervention Attainment	CR practitioner	49	8.18 (1.82)	2–10	11	6.91 (2.74)	2–10
BGSI-S Change in Attainment	Person with dementia	49	4.19 (2.81)	-1–9	12	2.58 (3.38)	-3–9
BGSI-S Change in Attainment	Carer	41	4.37 (2.82)	-2–9	10	3.30 (2.87)	0–9
BGSI-S Change in Attainment	CR practitioner	49	4.80 (2.48)	0–9	11	2.82 (3.25)	-2–9
		N (%)			N (%*)		
Level of goal attainment	CR practitioner	*n* = 49			*n* = 11		
0%		0 (0)			1 (9.1)		
25%		2 (4.1)			2 (19.2)		
50%		5 (10.2)			1 (9.1)		
75%		12 (24.5)			4 (36.3)		
100%		30 (61.2)			3 (27.3)		
(b) Classification of rehabilitation goals, based on categories developed in the GREAT trial							
		Goal 1 *n* (%)			Goal 2 *n* (%)*		
Using appliances, devices and the internet		15 (27.8)			7 (13.0)		
Managing everyday activities, tasks and situations		10 (18.5)			5 (9.3)		
Recognising, identifying, and naming		10 (18.5)			1 (1.9)		
Engaging in activities and personal projects		8 (14.8)					
Knowing what is happening		5 (9.3)					
Retaining or keeping track of information and events		4 (7.4)			1 (1.9)		
Locating belongings		1 (1.9)					
Managing emotions		1 (1.9)					
Keeping in contact and staying engaged with family and friends					1 (1.9)		
(c) Satisfaction with the intervention as indicated by survey responses from people with dementia and carers							
		People with dementia, n (%)			Carers, n (%)		
Did you find the GREAT CR sessions useful?		*n* = 41			*n* = 35		
Yes, very useful		32 (76.2)			22 (62.9)		
Yes, rather useful		7 (16.7)			13 (37.1)		
Did not make much difference		2 (4.8)			0		
No, not useful at all		0			0		
Would you recommend GREAT CR sessions to other people with memory difficulties or to families affected by memory difficulties?		*n* = 42			*n* = 35		
Yes		41 (97.6)			34 (97.1)		
No		0			1 (2.9)		
Not sure		1 (2.4)			0		

*SD* = *standard deviation. Changes in attainment scores were calculated as Post-intervention ratings minus Initial ratings, with a higher score indicating greater improvement. Bangor Goal-Setting Interview – Short Version, BGSI-S*

^*^percentages relate to the entire sample of people with dementia (n = *54); 39 of them (72%) had no Goal 2 set*

*Two of the goals had two components each and only the first ones were included in the classification above (one in ‘Managing*

*everyday activities, tasks and situation’ and one in ‘Recognising, identifying, and naming’). The other two components were both in*

*‘Using appliances, devices and the internet’ category*

*Cognitive rehabilitation, CR*

### Evaluation of costs

The practitioners in this study were primarily OTs employed in the NHS on a standard pay and grading structure. We calculated the per-person cost of NHS OTs delivering a course of GREAT CR consisting of six one-hour sessions under our implementation strategy. As we observed different combinations of input by qualified OTs and OT support workers we calculated costs for four skill-mix scenarios. As the time and costs of travel to conduct home visits are likely to differ locally, we present results both including and excluding travel costs. The scenarios, assumptions, calculation of unit costs and cost estimates are summarised in Table 3. Excluding travel time, the total cost of six sessions provided by a qualified OT was £349, which reduced to £239 if the first and last sessions were provided jointly with an OT assistant who conducted the intervening four sessions.

**Table 3:** Per-person cost estimates for a six-session course of CR under four staff skill-mix scenarios

Parameters	Basis for calculations							
(a) Scenarios and assumptions								
Skill-mix scenarios	1, A qualified OT provides all 6 sessions 2. A qualified OT provides the first and last sessions and an OT support worker provides the intervening 4 sessions 3. A qualified OT and an OT support worker jointly provide the first and last sessions and the OT support worker provides the intervening 4 sessions 4. A qualified OT and OT support worker jointly provide the first session and the OT support worker provides the remaining 5 sessions							
Staff grades	Qualified OTs are on NHS Agenda for Change Band 6 (‘Occupational therapist specialist’) [41] Unqualified OT support workers^ are either OT Assistants on Band 3 (‘Clinical support worker, higher level, OT’) or OT Technicians on Band 4 (‘OT technician’) [42]							
Training	20 h of staff time were allocated for training. Training was assumed to be accessed free of charge							
Supervision	Supervision was assumed to be provided in-house as part of routine staff supervision							
Session duration and preparation time	Sessions last 60 min. Preparation is estimated at 12 min per session, based on 12.5 min recorded in the GREAT trial							
Travel time	We could not identify any national statistics on average distance travelled for home visits; we therefore based our estimates on a published evaluation from an English NHS mental health Trust which reported that travel to home visits involved an average distance of 14 miles and took 25 min [43]							
Total time	Practitioner time per session was estimated at 72 min excluding travel time and 97 min including travel time, rounded up to 100 min							
(b) Calculation of unit costs								
Cost category	Unit			Cost per unit (£, 2019/20)		Source; notes on calculations		
OT Band 6—time	Year Hour			77 199 48.32		PSSRU UC, Table 9[44]		
OT Technician Band 4—time	Year Hour			50 659 31.27		PSSRU UC, Table 9[44]		
OT Assistant Band 3—time	Year Hour			38 720 25.02		PSSRU UC, calculated from healthcare assistants and other support staff annual earnings, Table 16.1, including salary on-costs (superannuation, employer’s NI), management costs (38.2% of salary costs), non-staff costs (24.5% of salary costs), capital costs (assumed same as for Band 4 in Table 9)		
OT Band 6—CR training	Annual equivalent			214.06		Based on 20 h staff time at the hourly cost of the practitioner. Annuitized over 5 years at 3.5%, assuming a refresher course would be needed after 5 years		
OT Tech Band 4—CR training				138.52				
OT Assistant Band 3—CR training				110.84				
OT Band 6 inc. training	Hour			48.46		Cost per hour of OT Band 6 * 1.0028		
OT Tech Band 4 inc. training				31.36		Cost per hour of OT Band 4 * 1.0027		
OT Assistant Band 3 inc. training				25.09		Cost per hour of OT Band 3 * 1.0029		
Mileage	Mile			0.36		NHS mileage allowances [45]		
(c) Cost estimates including and excluding staff travel to conduct home visits								
Scenario	1	2	3	4	1	2	3	4
Travel costs	Included				Excluded			
OT costs	500	167	83	167	349	116	58	116
OT Assistant costs	0	182	274	274	0	120	181	181
OT Technician costs	0	223	334	334	0	151	226	226
Total costs								
OT only	500	-	-	-	349	-	-	-
OT & OT Assistant	-	349	357	440	-	237	239	297
OT & OT Technician	-	390	418	501	-	267	284	342

National Health Service, NHS; Occupational Therapist, OT

Occupational Therapist, OT; OT Technician, OT Tech; Personal Social Services Research Unit, Unit Costs, PSSRU UC

Occupational Therapist, OT

### Further developments

In the later stages of the project, we undertook further development work to support sustainability and scaling-up. To promote awareness of the intervention we commissioned a short explanatory animation suitable for diverse audiences [31]. To make the approach accessible to people with dementia, we worked with a group of nine people living with dementia to co-produce a self-management guide based on CR principles which could be used either as a stand-alone tool or following a course of GREAT CR. The resulting resource, *My Life, My Goals*, can be accessed and downloaded via the Living with Dementia Toolkit [32] or the Alzheimer’s Society website [33].

The availability of sufficient practitioners with the skills and experience to provide GREAT CR is essential to future implementation. We developed an online version of the foundation-level training and trained 31 more practitioners. These were 23 additional practitioners from the partner organisations, and eight practitioners from other organisations. Five of the 31 practitioners completed all stages of the foundation-level training and one progressed to advanced-level training. We adapted the foundation-level training course into an e-learning format, incorporating educational videos prepared in collaboration with NHS Education for Scotland, and made this available to NHS practitioners via the NHS Learning Hub and to others via the GREAT CR website [31]. We also explored prospects for sustaining the community of practice. As most practitioners were OTs, we developed a dedicated special interest group within Royal College of Occupational Therapy structures.

## Discussion

This translational implementation project is one of few focusing on provision of home-based rehabilitative interventions for people with dementia. We aimed to develop a foundation for wider roll-out of an evidence-based personalised cognitive rehabilitation intervention for people with mild-to-moderate dementia. The implementation in community health and social care services in England and Wales was partially disrupted due to the COVID-19 pandemic, but six NHS organisations completed at least half of the 12-month implementation period. All six developed a small group of practitioners who successfully provided the intervention, although none reached their full targets for either the number of practitioners delivering, or the number of service users receiving, the intervention. The main barriers encountered were organisational, and the commitment of the managers and clinical leads who championed the intervention was key in overcoming or mitigating these. When delivered as part of routine services by trained NHS practitioners, mainly OTs, the intervention appeared at least as effective as under trial conditions. The degree of improvement (4.20 points for people with dementia, 4.37 for carers and 4.80 for practitioners) was greater than that seen in the GREAT trial where the corresponding indicators were 2.89, 3.17 and 3.61 points respectively [11], and the cost was relatively modest. The intervention was viewed positively by people with dementia, carers, practitioners and service managers. Not only did functioning in relation to the targeted goals improve, but people with dementia and carers also described wider gains such as increased confidence and independence. Findings suggested that further implementation would be beneficial for people with dementia and carers, and feasible provided organisational barriers are sufficiently addressed, and offered a basis for future scaling-up. However, implementation was unlikely to be sustained without ongoing funding and resourcing.

According to a scoping review, implementation initiatives in dementia care remain relatively limited [34]. The majority focus on residential long-term care, with few relating to implementation of personalised home-based interventions in community services. However, a small but growing body of evidence is emerging on implementation of community-based dyadic interventions utilising rehabilitation principles that aim to address the needs of both carer and care recipient and have a strong focus on supporting and working through carers. Our study adds the first example of implementing a rehabilitative intervention where the primary recipient is the person with early stage, mild-to-moderate dementia. The most important point arising from these studies, taken together, is that evidence-based rehabilitative interventions are effective when provided as part of community-based health services, and possibly even more so than when provided under trial conditions. Our findings in this respect are consistent with reports from dyadic interventions [35, 36]. In a related proof-of-concept study [37] we introduced the intervention into a group of four residential care homes, and results from 30 residents provide evidence that CR can be applied effectively in these settings, with residents potentially benefitting to a similar degree to those living in their own homes. This underlines the importance of translational efforts to make psychosocial interventions of this kind available to all those who could benefit.

As capability to implement an intervention will be reduced if it is perceived or experienced as too complex, adaptations are likely to be needed during the translation phase [36, 38]. In the case of GREAT CR, the number of sessions was reduced relative to the original trial protocol [11], but this resulted in a clearer focus on the primary outcome of goal attainment which showed improvements in the trial. We were able to introduce this change with some confidence based on feasibility work undertaken in the final stages of the trial and on the results of the original pilot trials where an eight-session protocol was used [13, 14], but this need for adaptation highlights the value of an early small-scale translational phase to build the foundation for future scaling-up [39].

Making rehabilitative interventions for people with dementia and carers widely available requires sufficient suitably trained and well-supported practitioners. Our findings show that practitioners respond positively to the opportunity to learn about and provide evidence-based interventions that they consider helpful to people living with dementia, consistent with findings from other programmes [35, 36]. However, the need for sufficient time and support to gain experience and confidence in delivering the intervention should not be underestimated. OTs are the group most likely to be involved in providing personalised rehabilitative interventions, but although the principles of such interventions align well with their professional training and values, they still require specific training and coaching, and confidence to manage the individual rather than prescriptive nature of the approach [35, 38]. The unexpected delay in getting started after initial training that we observed, also found in other studies [35], could reflect a need for role models or difficulty identifying suitable clients, or both. This was important to address and asking practitioners to identify suitable clients prior to initial training appeared to be a helpful strategy.

In our study as in others, and according to a scoping review not unusually for implementation studies in dementia care [34], the greatest barriers were organisational. This review emphasised that, throughout the evidence base, organisational factors are repeatedly cited as the main barrier to effective implementation, and reports of inadequate management support or insufficient time to complete heavy workloads are common. Furthermore, increased demand on services can tend to outstrip efforts to improve quality of care. Organisational priorities are subject to external influence and resourcing, reflected in our study by the types of service that health and social care commissioners in each area were willing to fund, and a common theme across different contexts and countries [35, 38]. Although we planned to work across both publicly-funded services and privately-run businesses, the organisations implementing the intervention were all NHS services. While there was enthusiasm for providing rehabilitative interventions with a more preventive focus, reflected in the rather ambitious targets set by some organisations, the services were mainly geared towards diagnosis and crisis management, and in some cases the culture did not embrace the value of psychosocial interventions. As in other studies [35, 38], it was challenging to establish referral pathways, highlighting the importance of effective communication within organisations and the need for thorough preparation and effective managerial and clinical leadership. Probably the biggest challenge in our study arose from the failure of participating organisations to allocate time for intervention delivery in practitioners’ workloads, despite initial agreement to do so. This has been observed also in other studies [38]. Ongoing resources and funding are needed if implementation gains are to be sustained [36].

Our study has several limitations. We proposed to explore implementation in three different types of community-based organisations but were only able to evaluate implementation in NHS services. Therefore, further work is needed to establish how the intervention could be integrated into other types of services and delivered by different staff groups. The reach of the implementation was curtailed by the COVID-19 pandemic, meaning that numbers were smaller than anticipated, and limiting the extent to which we could build on learning from Wave 1 during Wave 2. Some limitations in the estimation of CR costs should be noted. Supervision time was assumed to stay the same with the addition of a new treatment approach, supervisors were assumed not to require additional training themselves, and we assumed that there were no costs to organisations of accessing foundation-level training. These assumptions could contribute to underestimating the costs of CR. However, base-case estimates were similar to those reported in the GREAT trial which included costs of CR-specific specialist supervision. Furthermore, estimation of costs relates to the NHS setting and costs might vary in other service contexts. A final comment relates to the dilemma inherent in researching implementation in real-world settings. Some studies report implementation projects conducted using randomised trial designs [36, 40]. We considered it essential to use a design that would interfere as little as possible with the normal processes of service delivery and opted for a light-touch evaluation. This meant that practitioners delivering the intervention were involved in collecting outcome data from people with dementia and carers with whom they had developed a relationship, which may have influenced goal attainment ratings [35], and outcome data was obtained from a lower proportion of practitioners and participants than would be anticipated in a trial, again leaving open the possibility of bias. However, even this light-touch approach was considered burdensome and off-putting by some practitioners, potential participants with dementia and carers, and impacted on willingness to be involved. Despite these limitations, the study demonstrates some important strengths including directly obtaining the views of people with dementia on the intervention and provides a basis for future scaling-up of the implementation to allow a wider group of people with dementia to benefit.

Involvement of people with dementia and carers was another strength of the study. The GREAT CR intervention has been developed over several years with involvement of people living with dementia and their families at each stage, from initial single case experimental designs through small pilot RCTs to the large GREAT trial. For this small-scale implementation project leading on from the main trial we involved two people with dementia as part of the study leadership team; they were co-applicants on the grant and part of the project management group, and they contributed in important ways. They helped with explaining GREAT CR to potential participants in an accessible way and made sure resources such as handouts and evaluation questionnaires were easy to use. Our funder linked three carers from its group of research volunteers with the project and they met with us regularly; their perspective helped to shape the evaluation and think about how the approach could be shared more widely. As the project progressed, we involved a co-production group of nine people living with dementia who took the time to understand GREAT CR, reflected on it in the light of their own experiences, and then worked to create the My Life, My Goals self-management resource [32, 33] that they felt would be useful for others facing similar challenges, either complementing a course of GREAT CR or as a stand-alone resource. This has already attracted considerable interest. Involving people with dementia and carers had a significant impact on the project, and co-producing resources took this to a different level by giving people with dementia control over the process and outcome.

## Conclusions

This study, focused on implementation of a home-based personalised cognitive rehabilitation intervention for people with mild-to-moderate dementia, adds to a small but growing set of findings indicating that when rehabilitative interventions are delivered as part of routine services outcomes are as good as, or better than, those obtained under trial conditions. This underlines the importance of making interventions like GREAT CR available to as many people with dementia as possible. Ensuring that these interventions are widely available, however, requires organisational prioritisation, ongoing funding and investment in appropriately trained practitioners with sufficient time to work in this way and with adequate support. Given the potential of rehabilitative interventions to empower people with mild-to-moderate dementia and carers to adjust to and manage life with the condition, and ultimately reduce the need for crisis-driven responses by services, there is a strong case for reorienting service priorities and investing in widespread implementation.

## Supplementary information

**Additional file 1.**

## Data Availability

The datasets generated and analysed in this study are available from the corresponding author on reasonable request. The evaluation was approved by Wales Research Ethics Committee 5, reference 18/WA/0217. Written informed consent was obtained from all study participants. The work was carried out in accordance with relevant guidelines and regulations.

## Abbreviations

BGSI-S: Bangor Goal-Setting Interview – Short Version
CORD-PD: Cognitive rehabilitation for Parkinson’s disease dementia pilot trial
CR: Cognitive Rehabilitation
GREAT: Goal-oriented cognitive rehabilitation in early-stage dementia: multi-centre single-blind randomised controlled trial
GREAT CR: GREAT Cognitive Rehabilitation
GREAT-iP: GREAT into Practice
NHS: National Health Service
NICE: National Institute for Health and Care Excellence
NIHR: National Institute for Health Research
OT: Occupational Therapist
