# Supplementary material for: Implementing a home-based personalised cognitive rehabilitation intervention for people with mild-to-moderate dementia: GREAT into Practice
Source: BMC Geriatr. 2023 Feb 13;23:93. doi: 10.1186/s12877-022-03705-0 (PMC9925212; doi:10.1186/s12877-022-03705-0)
Supplement: Supplementary file 1 — Additional file 1. [file 12877_2022_3705_MOESM1_ESM.docx]

**Implementing a home-based personalised cognitive rehabilitation intervention for people with mild-to-moderate dementia: GREAT into Practice**

Linda Clare, Aleksandra Kudlicka, Rachel Collins, Suzannah Evans, Jackie Pool, Cate Henderson, Martin Knapp, Rachael Litherland, Jan Oyebode & Bob Woods

**Supplementary material**

Additional Text 1. Implementation strategy

Additional Table 1. Summary of the GREAT-iP implementation framework

Additional Text 2. Resources to support the implementation

Additional Text 3. Interview topic guides

Additional Table 2. Characteristics of people with dementia and carers completing a course of GREAT CR

Additional Table 3. Summary of findings from senior staff interviews

Additional Table 4. Characteristics and responses of CR practitioners completing the online survey

Additional Figure 1. CR practitioner responses to the closed questions in the online survey

Additional Table 5. Content analysis of CR practitioner responses to open-ended survey items

Additional Table 6. Summary of findings from CR practitioner interviews

Additional Table 7. Content analysis of responses to open-ended questions by people with dementia and carers

**Additional Text 1. Implementation strategy**

The implementation strategy covered the five domains of the Knowledge-into-Action Process Framework [14]: evidence, context, methods, adoption and outcome. Table 1 summarises, for each domain, the key elements to be addressed and the underpinning theoretical models. To operationalise the strategy, we developed a structured implementation planning tool and accompanying guidance for use with senior decision-makers and other stakeholders in each organisation. The tool was to be used collaboratively to understand the nature of the organisation and the specific service(s) that would be offering CR, identify key individuals to form a steering group to lead the implementation locally, discuss barriers and facilitators, suggest solutions to obstacles that could be foreseen, and specify targets including number of practitioners to be trained, number of people to receive CR, and any organisation-specific goals.

Each partner organisation was asked to identify a key decision-maker such as a senior manager to take leadership of the implementation and a local CR lead with relevant clinical experience to act as champion and influencer, and to either set up a local steering group to support the implementation or identify an existing oversight group that could carry out this role. Following a meeting between the steering group and a member of the project team, a tailored implementation plan was developed collaboratively using the implementation planning tool, and formally agreed. The necessity of allocating sufficient time for staff to undertake the intervention work and participate in supervision was emphasised. Each organisation was assigned a member of the project team as an external facilitator who would train and supervise staff, advise on implementation processes, and help to problem-solve regarding any barriers encountered.

Additional Table 1. Summary of the GREAT-iP implementation framework

| KTA Domains | Underpinning models | Implementation actions |
| --- | --- | --- |
| Evidence | PARiHS OMRU | Review and understand the nature and quality of the evidence for CR, and the type of innovation involved  Consider the way in which the research evidence is presented and how to ensure it is appealing to practitioners |
| Context | PARiHS OMRU Stetler | Understand the services within which implementation occurs, in terms of remit, culture, and leadership, and resources available  Understand the practitioners delivering CR, in terms of skills, knowledge, attitudes and existing practice |
| Methods | PARiHS OMRU Stetler | Use internal and external facilitation  Tailor implementation strategies to fit the context  Manage barriers |
| Adoption | OMRU Stetler | Explore the intent to deliver, and actual delivery of, the intervention  Examine how practitioners evaluate the intervention |
| Outcome | OMRU Proctor | Identify outcomes for practitioners, people with dementia, and carers  Quantify costs  Explore potential sustainability |

*Promoting Action on Research Implementation in Health Services, PARiHS; Ottawa Model of Research Use, OMRU; Stetler Model of Research Utilization, Stetler; Taxonomy of Implementation Outcomes, Proctor*

**Additional Text 2. Resources to support the implementation**

To support the implementation, we adapted and further developed resources prepared for or used in the GREAT trial.

Resources to increase awareness and engagement

We prepared leaflets explaining the approach for people with dementia and carers, health and social care professionals, and managers, and one of our experts by experience recorded a video describing her experience of working with a CR practitioner.

Practitioner training

We developed a training pathway for practitioners. A two-day foundation-level practitioner training course equipped practitioners to provide the intervention. Practitioners who provided CR to at least two people with dementia and prepared a satisfactory case report detailing one of these interventions became GREAT CR Practitioners eligible for advanced-level training. A half-day advanced-level training course equipped experienced practitioners to provide peer-support to colleagues delivering the intervention within their organisation, and on submission of a satisfactory case report demonstrating application of these skills, they became GREAT CR Advanced Practitioners. They were then eligible to undertake a train-the-trainer course, preparing them to facilitate the foundation-level training course using the materials provided.

Resources to support intervention delivery

We prepared a range of resources for practitioners: a practitioner handbook; a booklet giving practical examples of addressing a range of therapy goals; and a set of materials for use in sessions with people with dementia and carers, including handouts and a personal ‘My CR plan’ record sheet that could be shared with family members or paid carers. We planned to establish a community of practice to enable sharing of experiences across organisations.

**Additional Text 3. Interview topic guides**

Topic guide for interviews with local steering group members

1. Please can you tell me about how things have gone with implementing GREAT Cognitive Rehabilitation (CR) in your organisation?
   1. What went well?
      1. What were the most successful aspects?
      2. Were there any other positive aspects?
   2. What went less well?
      1. What were the biggest challenges?
      2. Were there any other difficulties?
   3. What, if anything, would you do differently if you started again, knowing what you know now?
2. What impact did the implementation of GREAT CR have on your organisation?
   1. What was the financial impact?
      1. What were the costs?
      2. Were there any savings made?
   2. What was the impact on the reputation of the organisation?
   3. What was the impact on your staff?
   4. What was the impact on the service users?
   5. At the start of the implementation process we completed an implementation plan and set some specific goals. Do you think these goals were achieved?
      1. If yes, what helped?
      2. If not, what were the barriers?
3. How sustainable do you think the changes in the organisation are?
   1. What has been done to encourage future use of GREAT CR at your organisation?
   2. Is there anything that is stopping the organisation from continuing to provide GREAT CR?
   3. What more could be done to encourage future use of GREAT CR?
4. What did you think of the implementation plan document?
   1. How useful did you find it for planning the implementation?
   2. What aspects were helpful?
   3. Were any aspects challenging?
   4. What, if anything, would you plan differently, knowing what you know now?
5. Suppose another organisation were interested in learning about GREAT CR, what would you say to them about:
   1. How easy or difficult it is to learn?
   2. How easy is it to provide to people with dementia?
   3. Any concerns you have about the approach?
   4. Which elements you think are most important?
6. Given the resources your organisation put into implementing CR, did you see the outcomes for service users that they expected?
7. How much would the CR service need to expand to become a mainstream service within your organisation? Would the organisation be able to fund that expansion?

Topic guide for interviews with CR practitioners

1. What was your personal experience of providing GREAT Cognitive Rehabilitation (CR)?
   1. Did you find GREAT CR useful in your work?
   2. What worked well?
      1. What were the most successful aspects?
      2. Were there any other positive aspects?
   3. What worked less well?
      1. What were the biggest challenges?
      2. Were there any other difficulties?
   4. Would you recommend GREAT CR to other individuals or families affected by dementia?
      1. If so, why?
      2. If not, why not?
   5. What, if anything, would you change about GREAT CR?
2. Please can you tell me about how things have gone with implementing GREAT CR in your organisation?
   1. What went well?
      1. What were the most successful aspects?
      2. Were there any other positive aspects?
   2. What went less well?
      1. What were the biggest challenges?
      2. Were there any other difficulties?
   3. What, if, anything, would you do differently if you started again knowing what you know now?
3. How sustainable do you think the changes in the organisation are?
   1. What has been done to encourage future use of GREAT CR at your organisation?
   2. Is there anything that is stopping the organisation from continuing to provide GREAT CR?
   3. What more could be done to encourage future use of GREAT CR?
4. Suppose another health professional were interested in learning about GREAT CR, what would you say to them about:
   1. How easy or difficult it is to learn?
   2. How easy is it to provide to people with dementia?
   3. Any concerns you have about the approach?
   4. What are the crucial elements that you think are most important?

Additional Table 2. Characteristics of people with dementia completing a course of GREAT CR, and carers where available

##### (a) People with dementia (n = 54)

|  | N (%) | Mean (SD) | Range |
| --- | --- | --- | --- |
| Age (n=48) |  | 76.31 (9.62) | 48-94 |
| Sex |  |  |  |
| Female | 24 (44.4) |  |  |
| Male | 30 (55.6) |  |  |
| Ethnic group |  |  |  |
| White British | 50 (92.0) |  |  |
| Asian / Asian British | 2 (3.7) |  |  |
| Black / African / Caribbean / Black British | 1 (1.9) |  |  |
| Other ethnic group | 1 (1.9) |  |  |
| Language (n=51) |  |  |  |
| English | 47 (87.0) |  |  |
| Welsh | 1 (1.9) |  |  |
| Other (Arabic, Punjabi, Urdu) | 3 (5.6) |  |  |
| Education |  |  |  |
| No formal qualifications | 16 (29.6) |  |  |
| GCSEs or equivalent | 9 (16.7) |  |  |
| Completed apprenticeship | 6 (11.1) |  |  |
| A levels or equivalent | 1 (1.9) |  |  |
| National Vocational Qualification | 1 (1.9) |  |  |
| Higher National Diploma | 4 (7.4) |  |  |
| Undergraduate degree | 9 (16.7) |  |  |
| Master’s degree | 4 (7.4) |  |  |
| PhD | 1 (1.9) |  |  |
| Other (Chartered Accountant; Higher education evening classes) | 3 (5.6) |  |  |
| Marital status |  |  |  |
| Married/re-married | 34 (63.0) |  |  |
| Widowed | 13 (24.1) |  |  |
| Separated/divorced | 6 (11.1) |  |  |
| Single | 1 (1.9) |  |  |
| Health (self-rating) |  |  |  |
| Very good | 19 (35.2) |  |  |
| Good | 14 (25.9) |  |  |
| Fair | 18 (33.3) |  |  |
| Poor | 3 (5.6) |  |  |
| Time since diagnosis of dementia (months) (n=47) |  | 12.70 (11.12) | 1-43 |
| Dementia type (n=51) |  |  |  |
| Alzheimer’s | 31 (57.4) |  |  |
| Vascular | 10 (18.5) |  |  |
| Mixed Alzheimer’s and vascular dementia | 9 (16.7) |  |  |
| Posterior cortical atrophy | 1 (1.9) |  |  |
| Global Deterioration Scale (n=51) |  |  |  |
| Level 1: No cognitive decline | 1 (1.9) |  |  |
| Level 2: Very mild cognitive decline (age assoc. memory impairment) | 1 (1.9) |  |  |
| Level 3: Mild cognitive decline (mild cognitive impairment) | 19 (35.2) |  |  |
| Level 4: Moderate cognitive decline (mild dementia) | 26 (48.1) |  |  |
| Level 5: Moderately severe cognitive decline (moderate dementia) | 4 (7.4) |  |  |
| Level 6: Severe cognitive decline (moderately severe dementia) | 0 |  |  |
| FAQ Self-rating (n=53) |  | 11.60 (8.31) | 0-33 |
| FAQ Rating by carer (n=38) |  | 16.08 (7.80) | 1-31 |

*N=54 unless specified otherwise; Cognitive Rehabilitation, CR; SD - standard deviation; GCSE – General Certificate of Secondary Education (age 16); A Level – Advanced Level qualification (age 18); FAQ - Functional Activities Questionnaire*

##### (b) Carers (n=41)

|  | N* (%) | Mean (SD) | Range |
| --- | --- | --- | --- |
| Age (n=38) |  | 67.89 (15.76) | 20-93 |
| Sex (n=41) |  |  |  |
| Female | 31 (75.6) |  |  |
| Male | 10 (24.4) |  |  |
| Ethnic group (n=40) |  |  |  |
| White British | 39 (97.5) |  |  |
| Asian / Asian British | 1 (2.5) |  |  |
| First language (n=38) |  |  |  |
| English | 35 (92.1) |  |  |
| Welsh | 3 (7.9) |  |  |
| Education (n=40) |  |  |  |
| No formal qualifications | 7 (17.5) |  |  |
| GCSE or equivalent | 12 (30) |  |  |
| A level or equivalent | 3 (7.5) |  |  |
| National Vocational Qualification | 2 (5) |  |  |
| Higher National Diploma | 1 (2.5) |  |  |
| Undergraduate degree (BA, BSc) | 3 (7.5) |  |  |
| Master’s degree (MA, MSc) | 3 (7.5) |  |  |
| PhD | 2 (5) |  |  |
| Other | 7 (17.5) |  |  |
| Relationship |  |  |  |
| Spouse | 26 (63.4) |  |  |
| Partner | 2 (4.9) |  |  |
| Son/daughter | 10 (24.4) |  |  |
| Friend | 1 (2.4) |  |  |
| Other | 2 (17.5) |  |  |
| Living arrangements |  |  |  |
| Does not live with the person with dementia | 8 (19.5) |  |  |
| Lives with the person with dementia | 33 (80.5) |  |  |
| Amount of help provided (n=32) |  |  |  |
| Provides no help in a typical day | 1 (3.1) |  |  |
| Less than 1 hour | 2 (6.3) |  |  |
| More than 1 hour and up to 2 hours | 5 (15.6) |  |  |
| More than 2 hours and up to 3 hours | 4 (12.5) |  |  |
| More than 3 hours and up to 5 hours | 3 (9.4) |  |  |
| More than 5 hours and up to 10 hours | 5 (15.6) |  |  |
| More than 10 hours but not overnight | 1 (3.1) |  |  |
| More than 10 hours including overnight | 10 (31.3) |  |  |
| Other | 1 (3.1) |  |  |
| Amount of help provided – hours per week (n=7) |  | 38.29 (58.11) | 4-168 |

*N=41 unless otherwise specified; SD - standard deviation; GCSE – General Certificate of Secondary Education (age 16); A Level – Advanced Level qualification (age 18)*

Additional Table 3. Summary of findings from senior staff interviews

| Theme | Brief description | Exemplar quotes |
| --- | --- | --- |
| Outcomes of implementation | All six interviewees listed various positive impacts of the project, while acknowledging that the outcome could have been better.  GREAT CR enriched the work of participating organisations by encouraging a more person-centred approach and creativity in the planning and delivery of interventions. These positive impacts on practice were thought to have led to better outcomes for people with dementia and their care partners, and they had translated into improved morale and confidence amongst staff. There were also indications that they were beginning to translate into system and process changes, for example through inclusion in research and audit discussions. There was no strong indication that implementation of GREAT CR had achieved an impact on participating organisations’ reputations amongst their peers or the public, but one interviewee felt that the organisation’s external reputation would have been damaged by act of omission had they not participated in GREAT-iP. | *“I think the staff loved it. They did the training and those clinicians really enjoyed it.” LSG008-05*  *“In terms of our team cohesion and our purpose in the roles we were in. It just strengthened, I think, our role confidence, I suppose I’d say. I think the language around our practice changed….I think previously… we would have identified that someone’s level of function or abilities had changed and that they weren’t doing things but I think we hadn’t thought too much, or enough, about the reasons for that as to whether or not that was to do with paying attention or I suppose the areas of cognitive difficulty. I think participating …took us back to really taking a closer look at why was someone having the problem?” LSG014-001*  *“[Carer] said how beneficial it had been because you get given a diagnosis, and then you're led to believe there is absolutely nothing you can do...Other than expect a decline in that person. And for her, it was important that she could see that there were lots of things that her husband could still do and achieve.” LSG009-001*  *“The other thing that went well that’s different is I… yeah, I think our experiences of working with care partners, I mean it really varied, but I think we all had experiences of working with care partners who were really, really enthusiastic and really learnt the techniques themselves, with our support.” LSG014-001* |
| Implementation processes | Lack of resources and protected time for delivering GREAT CR was a particular barrier, especially when combined with disruptive organisational changes such as reconfiguration of teams, operating models and care pathways. High numbers of vacancies and difficulty recruiting staff in implementing teams also served to constrain the number of individual GREAT CR programmes it was possible to deliver. One consequence of these difficulties was that implementation of GREAT CR had often been over-reliant on a single person or small group of ‘pathfinder’ staff. The dominance of a medical perspective and sometimes nihilistic views about dementia contributed to difficulties in setting up referral systems in some organisations and was described as a barrier to securing funding for psychosocial interventions in a broader context. | *“Our services are currently only commissioned or mainly commissioned to provide a very narrow service based on diagnosis and medical and medication follow-up. So I think that was something that was challenging for us…. I think one of the issues is that we trained clinicians that are too high up in the hierarchy. A lot of us got called away on a lot of other tasks… Something we couldn’t have predicted is that things changed in our service. That made it more difficult to identify people.” LSG004-11*  *“We had some colleagues who just weren’t interested but that… Just that they made some assumptions based on their own ignorance that it wouldn’t work for somebody or it wasn’t worth trying for somebody.” LSG014-001*  *“It took some time to get senior managers interested in or to make them aware of this approach that we wanted to put to them as a viable option for people with dementia after the diagnosis…. Although the managers accepted they needed the time to do the work and they agreed to it, my colleagues’ time wasn’t protected. There was a disconnect between the managers and what was actually happening in the team.” LSG008-05*  *“There has been a real sense of enthusiasm to do something more for people with dementia but the actual practicalities of recruiting people, having clinicians available to do the work, that has been the issue. We have struggled to find time for the clinicians to actually provide it. There have been some systemic reasons why it has been hard to transfer that enthusiasm to actual interactions in a clinical setting…We have managed to achieve it, we have managed to keep it going despite all of the hurdles.”*  *LSG008-09*  *“There was a... a lot of goodwill to start the project. But in reality, you know, there were some difficulties and some challenges, and obviously COVID just threw it totally off.” LSG011-013* |
| Prospects for sustainability | All six interviewees expressed enthusiasm and motivation to continue GREAT CR provision within their organisations and some described arrangements for taking the work forward, although they were mostly opportunistic plans for continuation by front line teams while attempts were being made to secure resources for more substantive arrangements. Interviewees indicated that the link to the original research and subsequent implementation programme had been helpful by raising awareness and lending legitimacy to GREAT CR as an intervention. One interviewee reported that the participation in GREAT-iP provided a firm base for implementation and the work undertaken resulted in a greater likelihood of CR becoming a feature of regular practice. There were questions over the degree of ownership among senior decision-makers in participating NHS Trusts and difficulties of challenging established service priorities specified by commissioners, such as early diagnosis and crisis support, to make room for cognitive rehabilitation. | *“It was just from, yeah, perhaps more from the sort of financial operational management side of the service at a senior level that we might not be able to continue afterwards and how that might be a problem.” LSG014-001*  *“Hopefully cognitive rehabilitation will…be seen as not a luxury…but as something we need to be offering as standard. But at the moment it is new, it is additional…They [NHS Trust] have asked us to come up with a business case for cognitive rehabilitation. I have been left with this to take forward, and the Trust haven't owned it yet in a sense.” LSG008-05*  *“I do think cognitive rehabilitation is a useful intervention and, as I mentioned before, I do think that the population, when they understand more about it and are more aware of it, they will be asking for it.” LSG008-09* |

Additional Table 4. Characteristics and responses of CR practitioners completing the online survey (n=24)

|  | **N (%)** | **Mean (SD)** | **Range** | | |
| --- | --- | --- | --- | --- | --- |
| Age |  | 45.21 (5.99) | 31-54 | | |
| Sex |  |  |  | | |
| Female | 21 (87.50) |  |  | | |
| Male | 2 (8.33) |  |  | | |
| Prefer not to say | 1 (4.17) |  |  | | |
| Ethnic group |  |  |  | | |
| White | 21 (87.50) |  |  | | |
| Mixed / Multiple ethnic groups | 2 (8.33) |  |  | | |
| Prefer not to say | 1 (4.17) |  |  | | |
| English as primary language | 24 (100) |  |  | | |
| Highest level of education achieved |  |  |  | | |
| Higher National Diploma or equivalent | 1 (4.17) |  |  | | |
| NVQ Level 5 | 1 (4.17) |  |  | | |
| Undergraduate degree or equivalent | 17 (70.83) |  |  | | |
| Master’s degree or equivalent | 3 (12.50) |  |  | | |
| PhD or equivalent | 2 (8.33) |  |  | | |
| Job title |  |  |  | | |
| Occupational therapist | 8 (33.33) |  |  | | |
| Specialist occupational therapist | 8 (33.33) |  |  | | |
| OT Technician | 1 (4.17) |  |  | | |
| Principal clinical psychologist | 1 (4.17) |  |  | | |
| Clinical psychologist | 1 (4.17) |  |  | | |
| Senior Dementia Practitioner | 2 (8.33) |  |  | | |
| Team Leader | 1 (4.17) |  |  | | |
| Support worker/healthcare assistant | 2 (8.33) |  |  | | |
| Completed previous training courses in the area of dementia | 18 (75.0) |  |  | | |
| Employment status - permanent position | 24 (100) |  |  | | |
| Hours per week worked in current job |  |  |  | | |
| 8-16 hours | 2 (8.33) |  |  | | |
| 17-24 hours | 6 (25.00 |  |  | | |
| 25-32 hours | 5 (20.83) |  |  | | |
| More than 32 hours | 11 (45.83) |  |  | | |
| Key responsibilities in relation to working with people with dementia and/or their carers | | |  |  |  |
| Formal assessment of needs and/or cognition | 23 (95.83) |  |  | | |
| Signposting/referrals to sources of support | 20 (83.33) |  |  | | |
| Providing information about dementia | 19 (79.16) |  |  | | |
| Emotional support | 18 (75) |  |  | | |
| Developing care plans | 18 (75) |  |  | | |
| Planning or overseeing the work of other staff members | 15 (62.5) |  |  | | |
| Assistance with daily tasks at the person’s home | 13 (54.16) |  |  | | |
| Providing company and assistance with leisure activities | 4 (16.66) |  |  | | |
| Other | 5 (20.83) |  |  | | |
| Satisfaction with current job |  |  |  | | |
| Very satisfied | 6 (25.00) |  |  | | |
| Satisfied | 18 (75.00) |  |  | | |
| Satisfaction with the quality of care provided to people with dementia in the job | | |  |  |  |
| Very satisfied | 6 (25.00) |  |  | | |
| Satisfied | 18 (75.00) |  |  | | |

*Cognitive Rehabilitation, CR*


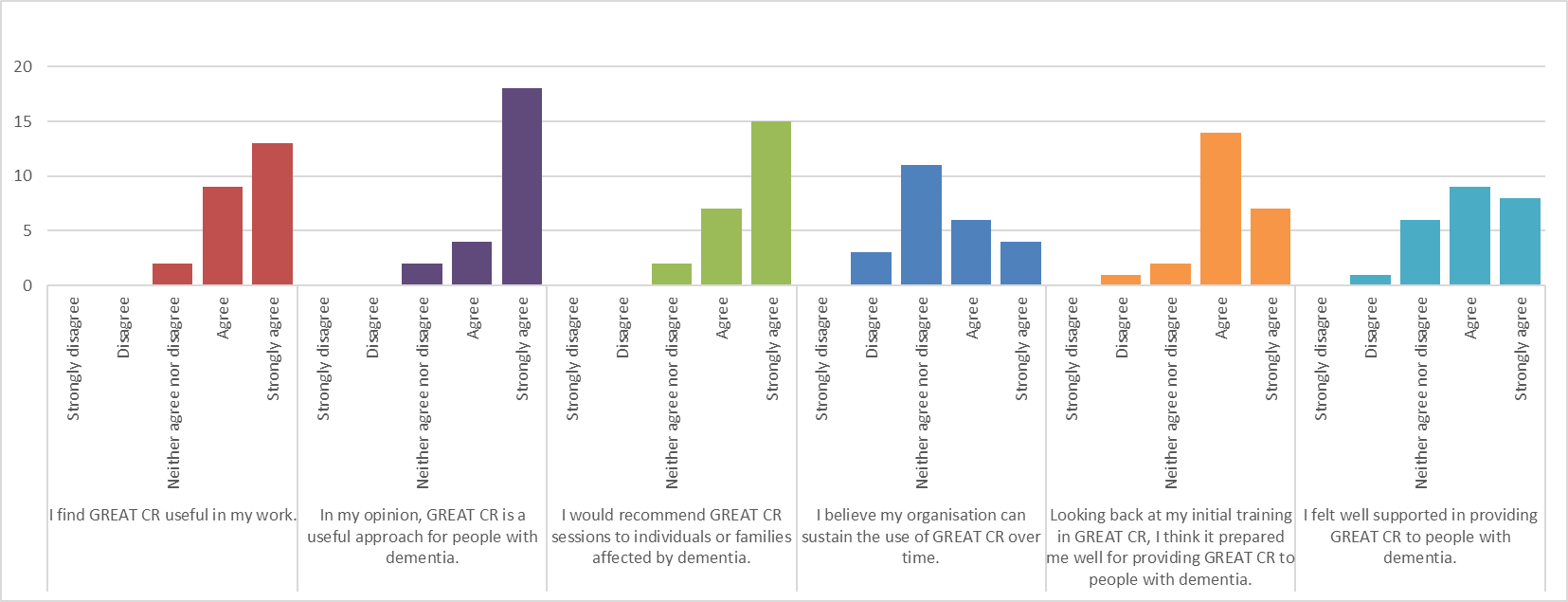


Additional Figure 1. Cognitive Rehabilitation practitioner responses to the closed questions in the online survey (n = 24)

Additional Table 5. Content analysis of CR practitioner responses to open-ended survey items

Summary of responses

| Category | Number of codes | % of all codes | % of practitioners contributing to the theme |
| --- | --- | --- | --- |
| Features of GREAT CR that worked particularly well (comments from 19 CR practitioners) | | | |
| Effective techniques | 13 | 36.1 | 68.4 |
| Person-centred, individualised and personal | 5 | 13.9 | 26.3 |
| Positive impact on participants | 5 | 13.9 | 26.3 |
| Promotes self-management in participants | 3 | 8.3 | 15.8 |
| Useful and relevant | 3 | 8.3 | 15.8 |
| Improved practitioner skill set | 3 | 8.3 | 15.8 |
| Rewarding to practitioners | 2 | 5.6 | 10.5 |
| Features of GREAT CR that proved less helpful (comments from 21 CR practitioners) | | | |
| Nothing | 5 | 22.7 | 23.8 |
| Some techniques less successful in certain settings | 4 | 18.2 | 19.0 |
| Prone to disruption in complex circumstances, i.e. illness | 4 | 18.2 | 19.0 |
| Research element of the project - too much paperwork | 3 | 13.6 | 14.3 |
| Unsuitable participants / recruitment pathways | 2 | 9.1 | 9.5 |
| Suboptimal involvement of carers | 2 | 9.1 | 9.5 |
| Limited experience with the intervention | 1 | 4.5 | 4.8 |
| Suggestions for improvement (comments from 18 CR practitioners)   - - - - 1. Training and/or supervision | | | |
| Provide follow-up or refresher training | 5 | 13.9 | 27.8 |
| More options for accessing support or supervision | 5 | 13.9 | 27.8 |
| More examples to draw from | 2 | 5.6 | 11.1 |
| Role-playing to practise elements of CR | 1 | 2.8 | 5.6 |
| Training could be condensed | 1 | 2.8 | 5.6 |
| - - - - 1. Other |  |  |  |
| More physical and/or financial resources including time | 6 | 16.7 | 33.3 |
| Target a more appropriate client group | 3 | 8.3 | 16.7 |
| Increase the number of professionals able to offer CR | 2 | 5.6 | 11.1 |
| Address uncertainty about long-term sustainability | 2 | 5.6 | 11.1 |
| - - - - 1. None needed |  |  |  |
| Positive opinion of GREAT CR | 5 | 13.9 | 27.8 |
| No improvements needed | 4 | 11.1 | 22.2 |

*Cognitive Rehabilitation, CR*

(b) Example quotes taken from practitioners’ anonymised survey responses

| Topic area | Example quote |
| --- | --- |
| Worked well | *“The person-centred approach means that it can make a big difference to daily life, as the goals are meaningful to the person.”* |
| Less helpful | *“Physical illness can easily disrupt any new learning or altered routine and make CR seem less effective… All features are helpful, depends on my skill in application and the person's interest in engaging.”* |
| Potential for improvement | *“Greater commitment should, perhaps, be sought from the Practitioner's organisation about scope and willingness to embed this approach in their service. This approach should be offered to all those people with dementia that are suitable as it's a powerful restorative experience for them.”* |

Additional Table 6. Summary of thematic analysis of data from CR practitioner interviews

| Theme | Description | Example quotes |
| --- | --- | --- |
| Application of GREAT Cognitive Rehabilitation (CR) | Practitioners were consistently positive about the intervention. The flexibility and adaptability of the approach was a frequently mentioned strength.  Practitioners gave detailed examples of how it benefitted people with dementia and carers. These were both immediate and specific, such as being able to learn co-workers’ names and hence stay in employment, and more general, such as restoring confidence, instilling a sense of hope, facilitating independence, and reducing the burden on carers.  Practitioners described positive benefits for themselves as professionals, including satisfaction about being able to focus on preventing problems and improving quality of life rather than reacting to crises.  Practitioners emphasised that successful application required sufficient time. | *“That it allowed creativity in my work, was brilliant. I loved it, and I still do, and if I had the chance I would do a lot more of it. CRP008=05It made a huge improvement in the couple’s relationship and that was a big hit for me, because it demonstrated people could see change quickly and so could I.”*  *CRP008-05*  *“Hopefully, we are providing a better service with the skills that we have acquired” CRP004-08* |
| Targeting of GREAT CR | CR was most effective when offered to people whose cognitive impairment was significant enough to create noticeable challenges that could be translated into therapy goals, and where the person recognised at least some of the difficulties and was prepared to invest time and effort to improve the situation.  Involvement of carers was important for success. Although ultimately intended to reduce burden, some viewed the intervention as additional work and resisted involvement. | *“Yes, definitely [would recommend GREAT CR to people with dementia]. I think as long as they have got enough ability and enough insight to be able to work with you, then yes, definitely.” CRP004-04* |
| Factors affecting delivery of GREAT CR | Developing confidence to deliver the intervention was crucial. This involved consolidating training through actual practice and having access to supervision and support as well as developing confidence in realistic goal setting, managing expectations and adapting programmes in response to changing circumstances.  Resource limitations made it difficult to devote the time needed to get to know the person and design a personalised intervention, and created conflict boundaries between providing CR and routine work.  A key organisational issue affecting implementation was identifying people with dementia who had the right profile of needs through referral and liaison systems. This required staff in the extended service to have a good understating of the CR to enable suitable referrals. | *“There was some confidence issues I would say, with the staff that were trained and actually using it. I don't know if that held people back from actually delivering it.” CRPO11-013*  *“Because it’s not seen as high priority as things like carers going into complete crisis, social breakdown (…) I know as a service the idea is that we are looking at people’s well-being, but sometimes I don’t know if I really feel that in my day to day job.” CRP001-05*  *“If people are being assessed and receive their diagnosis and start to get signposted at the same time, in the space of one and a half to two hours, there is very little time, at that point, to also think about referral for rehabilitation” CRP004-11* |
| Sustainability of GREAT CR | There was an intention to continue to provide GREAT CR, but no specific plans.  Most services appeared to focus on diagnosis and on crisis management for people with more advanced dementia, making it difficult to incorporate a preventative well-being intervention.  Involving ancillary staff could support sustainability but would need to be overseen by qualified professionals to avoid loss of quality.  There was a need for commissioners and senior managers to build GREAT CR into service specifications and operating models.  Some practitioners aimed to incorporate CR within their own clinical practice independent of formal organisational initiatives. | *“I think what we’re almost waiting for is a higher level decision about what's going to happen, and that requires quite a lot of input and motivation, doesn’t it, to keep moving it on? To keep it in the forefront of people’s mind, keep it live. The trouble is other things get prioritised, other things come in. It can get a little bit lost.” CRP001-05* |

Additional Table 7. Content analysis of responses to open-ended questions by people with dementia and carers

1. Responses from people with dementia (N = 41)

| Topic areas and categories | Number of codes | % of all codes | % of  PwD |
| --- | --- | --- | --- |
| What was helpful about the GREAT CR sessions? | | | |
| Learning and relearning skills and strategies | 28 | 43.8 | 34.2 |
| Having an appreciation of the external input and effort | 10 | 15.6 | 17.1 |
| The social interaction | 7 | 10.9 | 9.8 |
| The GREAT CR sessions (e.g. structure) | 6 | 9.4 | 7.3 |
| It provided wider personal benefits | 5 | 7.8 | 2.4 |
| It is helpful in the right circumstances | 3 | 4.7 | 7.3 |
| It was useful for learning about help available | 2 | 3.1 | 4.9 |
| It provided reassurance about own skills or strategies | 2 | 3.1 | 4.9 |
| Allowed for acknowledgment of the challenges ahead | 1 | 1.6 | 2.4 |
| What was less helpful about the GREAT CR sessions? | | | |
| Nothing considered unhelpful | 14 | 63.6 | 34.2 |
| Participant had concerns due to having dementia | 4 | 18.2 | 12.2 |
| Having more time and/or sessions would have been beneficial | 2 | 9.1 | 7.3 |
| Found GREAT CR sessions to be inappropriate or irrelevant | 2 | 9.1 | 4.9 |
| What difference (if any) have GREAT CR sessions made to your daily life? | | | |
| Provided an opportunity to learn or improve knowledge and abilities | 19 | 34.6 | 24.4 |
| Allowed an opportunity to increase levels of confidence | 11 | 20.0 | 14.6 |
| Had the added benefit of improving wider physical or mental health | 5 | 9.1 | 4.9 |
| Increased the level of awareness (of condition and limitations) | 4 | 7.2 | 7.3 |
| Increased the opportunity to engage in hobbies and interests | 3 | 5.5 | 4.9 |
| Allowed the opportunity for increased enjoyment in undertaking hobbies and interests | 3 | 5.5 | 4.9 |
| Provided an opportunity to make contact with a professional | 3 | 5.5 | 7.3 |
| Allowed an opportunity to increase independence | 3 | 5.5 | 2.4 |
| Improved the ability for increasing social engagement | 2 | 3.6 | 4.9 |
| Unsure of benefit | 2 | 3.6 | 2.4 |

*Person with dementia, PwD; Cognitive Rehabilitation, CR. One person with dementia did not provide any responses to the open-ended questions*

1. Example quotes from people with dementia

| Topic area | Example quote |
| --- | --- |
| What was helpful | *“The sessions were hands on and emphasized the things I needed to do my daily tasks and feel more confident.” P8341*  *“I didn't know how to use my mobile phone and now I do.” P4371*  *“Very enjoyable, had fun and made me feel helpful.” P7281* |
| What was less helpful | *“Nothing whatsoever!” P4211*  *“Could come more often!” P4621*  *“In the beginning worried about not being able to learn.” P7011*  *“I have to go back to the fact that I have no memory.” P1161* |
| What difference did CR make | *“More confidence. Boosted my feelings about myself. More secure.” P1161*  *“Using the method I can remember names of work colleagues.” P1151*  *“I have become more independent and will now go out on my own regularly. I have attempted volunteering in a charity shop and will now get shopping on my own.” P4371* |

*Cognitive Rehabilitation, CR*

1. Responses from carers (N = 35)

| Topic areas and categories | Number of codes | % of all codes | % of  carers |
| --- | --- | --- | --- |
| What was helpful about the GREAT CR sessions? | | | |
| Different skills, strategies and techniques to support PwD were learnt | 20 | 29.9 | 51.4 |
| The PwD successfully achieved the specific goal(s) | 11 | 16.4 | 28.6 |
| GREAT CR led to wider personal implications for the PwD | 10 | 14.9 | 25.7 |
| An appreciation of the therapist’s knowledge and manner | 7 | 10.5 | 20.0 |
| An appreciation of being able to talk to therapist | 7 | 10.5 | 14.3 |
| GREAT CR is a useful and appropriate technique | 5 | 7.5 | 14.3 |
| GREAT CR led to wider personal implications on the carer | 5 | 7.5 | 14.3 |
| It provided affirmation of doing the right thing | 2 | 3.0 | 5.7 |
| What was less helpful about the GREAT CR sessions? | | | |
| Nothing considered unhelpful | 21 | 65.6 | 60.0 |
| Suggested improvement in the GREAT CR content or resources | 3 | 9.4 | 8.6 |
| GREAT CR not appropriate for time or situation | 3 | 9.4 | 8.6 |
| Lack of financial support | 2 | 6.3 | 5.7 |
| Some aspects of daily functioning did not improve | 2 | 6.3 | 5.7 |
| Caused an odd argument | 1 | 3.1 | 2.9 |
| What difference (if any) have GREAT CR sessions made to your daily life? | | | |
| GREAT CR has increased the level of confidence in the PwD | 12 | 23.1 | 34.3 |
| The PwD has developed or learnt new skills | 10 | 19.2 | 28.6 |
| The carer has developed or learnt new skills to support the PwD | 6 | 11.5 | 17.1 |
| Helped develop a more positive future outlook for carer and/or PwD | 6 | 11.5 | 14.3 |
| GREAT CR has had a positive impact on the carer | 5 | 9.6 | 14.3 |
| The PwD is undertaking hobbies and interests | 4 | 7.7 | 11.4 |
| GREAT CR provided a sense of achievement | 4 | 7.7 | 11.4 |
| GREAT CR provided another source of information | 3 | 3.8 | 5.7 |
| Uncertain or no difference stated | 2 | 4.7 | 5.7 |

*Person with dementia, PwD; Cognitive Rehabilitation, CR*

1. Example quotes from carers

| Topic area | Example quote |
| --- | --- |
| What was helpful | *“My husband began to remember names.” C8382*  *“It helped my husband to believe that he could have some control over his memory loss. It gave him a focus and a chance to try and retain some control and skills, and to maintain some independence.” C9222*  *“It has brought my wife out of herself, more confident with the phone. Before she wouldn't answer the phone at all - now she does. I seriously think it would help others and benefit them.” C9342* |
| What was less helpful | *“Nothing. It was all helpful.” C8352*  *“It would be more useful for someone who has a family member close by to give regular repetition of the suggestions made by the OT.” C4152*  *“It was too short!!! Mum would really benefit from ONGOING sessions like this.” C1222* |
| What difference did CR make | *“Taught me how to cope with my husband's memory loss more patiently!” C4622*  *“[Mother] is much more confident in planning daily tasks and finding information. This has taken pressure off me as her first point of contact.” C8362*  *“Boosted confidence. Started doing things/activities on own initiative without prompting. Very beneficial.” C1222*  *“A big difference - A good outcome by realising there are different strategies to manage the problems we face which do help. Just finding the right strategy and implementing them.” C1072* |

*Occupational Therapist, OT*
